# The Role of Recurrence Plots in Characterizing the Output-Unemployment Relationship: An Analysis

**DOI:** 10.1371/journal.pone.0056767

**Published:** 2013-02-27

**Authors:** Petre Caraiani, Emmanuel Haven

**Affiliations:** 1 Institute for Economic Forecasting, Romanian Academy, Bucharest, Romania; 2 School of Management and Institute of Finance, University of Leicester, Leicester, United Kingdom; Humboldt University, Germany

## Abstract

We analyse the output-unemployment relationship using an approach based on cross-recurrence plots and quantitative recurrence analysis. We use post-war period quarterly U.S. data. The results obtained show the emergence of a complex and interesting relationship.

## Introduction

A key relationship in macroeconomics is the one between unemployment and output. This relationship is of prime importance from a purely pragmatic point of view: it is of a great interest to know how to model the way unemployment changes when a recession hits the economy and also how fast unemployment will recover. The policy implications of the relationship between output and unemployment are extremely important: they steer the longevity of an economy and of its government (for instance how viable is expansionary output in the future) and they (hopefully) make sure that changed levels of output can translate in better levels of employment. As we know, from casual inspection, i.e. by simply looking at today's economy, it is far from obvious what the precise relationship is between output and employment. Hence, studying it further and trying to characterize the relationship, via recurrence plots, as we attempt to do in this paper, can be an important mission. We believe that in this paper we are able to isolate patterns in the dynamics of unemployment and output which, we think, could not be otherwise isolated using standard econometric tools.

In macroeconomics the so called ‘Okun's Law’ refers to the regularity observed in the relationship between the changes in production (or the GDP of a country) and its unemployment rate. By ‘output’ we mean production or GDP (in economics, those terms are used in economics interchangeably). Friedman and Wachter [1] define Okun's law [2] as a fixed relationship between levels of unemployment and levels of the output gap. With the ‘gap’ is meant the difference between actual output and so called ‘potential output’ which is the level of output occurring at the natural rate of unemployment. Such a level of unemployment appears when all unemployment is purely of a ‘voluntary’ type.

Initially, the two variables (i.e. unemployment and output) were estimated with the use of a simple linear regression. However, [1] remark the relationship in early papers, was estimated with the simplifying assumption that the trend rate of change of potential output would be i) exogenously given and ii) steady. In their own paper, [1] relax those two assumptions and many other economists since then have investigated other ways of improving on the original approach to Okun's law. Nalewaik, Diebold and Landefeld [3] point out that since the 1980's Okun's law has taken on a different character. They specifically mention that unemployment has become more responsive to changes in output.

The importance of this relationship is underscored by its link with the so called ‘Phillips curve’. In the Keynesian model, an increase in aggregate demand beyond the point of potential output will lead to very high levels of inflation. This is due to the fact that the aggregate supply curve becomes very steeply sloped close to potential output. The so called short run ‘Phillips curve’ [4] attempts to show that a higher (lower) rate of inflation is accompanied by a lower (higher) rate of unemployment. However, in the late 1960's, the Phillips curve became contested [1], [5], [6], [7], [8]. The expectations augmented Phillips curve which emerged out of Friedman and Phelps' work seems to have a close connection to the so called ‘New Keynesian economics’ [9], [10], [11].

This paper does not want to pretend to delve further into the economic intricacies of new Keynesian economics. Instead we want to focus on proposing a new approach to explain nonlinearities in the two key variables we mentioned at the beginning of this introduction. The increased awareness of the existence of nonlinearities in economics has surely influenced the way economists model the relationship between output and unemployment. Contributions have started to focus more on asymmetries and nonlinear dynamics mainly because it has been established that the dynamics of unemployment in a period of economic growth differ from those in a recession [12], [13], [14], [15]. However, as is (still) the case with most macroeconomic subjects, this topic has not yet been studied from the perspective of new nonlinear techniques from physics.

Hence, in this paper, we propose a re-evaluation of this key relationship using concepts and techniques from physics, like determinism measures and recurrence plots. Our approach is based on cross and quantitative recurrence analysis. Those methodologies will be presented in the paper.

We believe the contributions of this paper are as follows.

Firstly, we analyse the underlying dynamics of unemployment and output using a novel approach which might lead to answers to well-known issues in the areas of nonlinearities and even determinism. We use recurrence plots and quantitative recurrence analysis to this end. Our findings may hopefully shed more light on whether business cycles are possibly characterized (or not) by a degree of determinism. This is a debate which is still not settled.

Secondly, we analyse the relationship between the time series on unemployment and output (GDP) using cross recurrence plots and again quantitative recurrence analysis. We believe that through this approach we can reveal some aspects of the relationship between unemployment and output that would otherwise be hidden with a standard econometric approach.

## Methods

### Basic motivation for the use of recurrence analysis in economics

Recurrence plots have been successfully used in the sciences for some time now and more recently it has found inroads in social science, notably in economics. We provide for relevant references below in the paper. We believe that the adoption of recurrence plots as a tool of analysis is based on the view that it is a useful methodology in understanding better the dynamics of single series or of relationships. We apply this approach to a topic that remains of very high interest to both the macroeconomics academic community and the general public: that is the relationship between production (or also output) and unemployment.

While initial studies focused on the linear aspect of the ‘output’ and ‘unemployment relationship’, there is more and more awareness the relationship between production and unemployment may well be nonlinear in nature. We think the approach proposed by us can better highlight this nonlinear relationship. The nonlinear relationship has deep implications on a macroeconomics level and this was already argued for in the 1960's. The paper by [16] on the nonlinear theory of the employment cycle, for instance argues for a Phillips curve with a loop effect (i.e. a nonlinear effect). However, the quest for uncovering nonlinearities in the above relationship did not produce a steady stream of papers, since the 1960's. As an example, it was only in 2001 that [15] argues for a nonlinear relationship and he indicates that such nonlinearities allow for a better explanation of the varying (in) effectiveness of macroeconomic policies which target unemployment. The paper by [17] discussed in much detail what the consequences can be of policy-induced macro-economic recessions. This is indeed an important consequence given the current economic climate but it is also of importance within the wider European Community context, as [15] indicates, such nonlinear relationships affect aggregation (from a country level to a multiplicity of countries level, such as with the European community). In the work of [18] it is shown that with the use of wavelets, one can test for the presence of lower output volatility in US output (since the late 1940's). This lower volatility [18] claims to be a consequence of changes in a dynamic process. We wonder if this ‘dynamic process’ has a link to what we will call in our paper the periods of ‘dynamic discontinuity’. In summary with the few references cited here, we attempt to show that there is scope to use this methodology to uncover the possible presence of nonlinearities in the relationship between unemployment and output.

Recurrence plot based techniques have been used to study the nonlinear relationships between different variables. The reference paper by Marwan and Kurths [19], introduces bivariate analysis (with an emphasis on nonlinearity) and it was also shown there that the bivariate recurrence plots can not only deal with linearity but also with the nonlinear aspect of nonlinearity. The possibility of using recurrence plots to pinpoint transitions which from an economics point of view are important is a key contribution from this methodology. As an example, the possibility of arguing for a transition to different states (what we call in our paper a ‘dynamic discontinuity’) during recessions is of great use. This characteristic that recurrence plots are very good at change point detection is highlighted in [20], [21].

### Theoretical background

In 1987 the term ‘recurrence plots’ was proposed in the paper by Eckmann et al. [22] and they emphasize the major benefit of using such plots: i.e. one can derive time information explicitly from a dynamical system.

In economics and finance, the methodology began to be applied many years after its inception in papers by [23], [24], [25], [26], [27], [28] and [29]. As one can appreciate the number of applications of this methodology in economics and finance is still small. It also needs to be said that most of those contributions focussed on a univariate based analysis.

We discuss in the paragraphs below the main points of this approach and we follow closely Marwan and Kurths [19] and Marwan [30].

The phase space trajectory dynamics can be represented as:



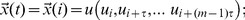
(1)where 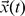 is the phase space trajectory; 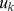 is a time series with 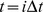, and 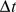 is the sampling time; 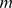
is the embedding dimension and 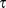
is the time delay.

There are different approaches in setting the parameters corresponding to the embedding dimension 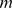 and the delay 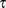. See [19] for some suggestions. One defines the recurrence plot based on the following formula:



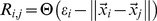
(2)


where 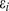 is a predefined parameter characterizing the distance between two neighbouring points; ∥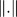 is the norm (normally the Euclidean norm is used); while 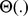 is a Heaviside type function.

Since in this paper, we are interested in the relationship between unemployment and output, we will, besides using classical recurrence plots, also use cross recurrence plots. Amongst the first authors to extend recurrence plots into cross recurrence plots were Zbilut, Giuliani and Webber [31]. Re-consider, equation (2) from above, but alter the ingredients in the norm:



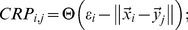
(3)


where 
and 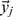 are respectively the reconstruction of the first and second series (different dimensions are possible) in the phase space. Again, we can visualize important features of the relationship between the two systems. Long diagonal lines indicate similar behaviour in the phase space. See [19] and [30].

Several measures derived from quantitative recurrence analysis will be used in this paper. Quantitative recurrence analysis is a further development of recurrence plots and is due to contributions from [32] and [33].

In what follows below, we present the definitions of the main complexity measures we will employ in this paper. We continue using [19] and [30]. We will also be using the univariate approach (as originally proposed in [32]).

The recurrence rate, 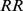, is defined as:



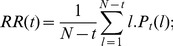
(4)


where 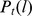 is the distribution of the diagonal line lengths (for a diagonal parallel to the main diagonal); 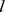 is the length of the line structure; 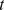 is an index of the relative location of the diagonal line; 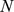
is the dimension of the vector 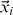. 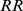
measures thus the density of recurrence points [30]. The 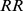 can also be used as an indicator of changes in the dynamical system.

We also consider the ‘determinism’ measure, 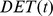 which is given by:



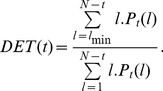
(5)


Low values indicate stochastic systems, while higher values are an indication of a degree of predictability. The intuition with this measure is more subtle. Although [30] indicates 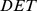
gives the predictability of the system, we need to keep in mind that predictability and determinism are not obvious equivalences. In fact [30] cautions about the reliability of the 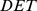
measure. The 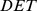
and 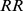
are examples of so called ‘Recurrence Quantification Analysis’ (RQA) (see [32]). RQA measures are statistical measures and [34] makes the important remark that estimating the confidence of such measures is still an open question. Consequently, the 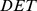
and 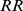
measures have not yet received a lot of attention as to their statistical robustness.

In the case of a bivariate analysis, another important measure is the average diagonal length, 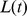:



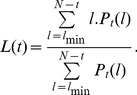
(6)


It indicates the degree of coincidence of two systems.

In the case of univariate analysis, a measure of entropy is used. The Shannon entropy, 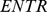, is:



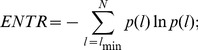
(7)


where 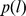 is defined in function of the cumulative distribution of the line length.

The 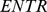 measure is also an example of Recurrence Quantification Analysis (RQA). 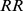, 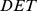
and 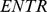 are measures which are based on diagonal lines. The laminarity and trapping time measures in equations (8) and (9) below follow [30] and are based principally on vertical lines in a recurrence plot. As [30] indicates, laminar states refer to ‘chaos-chaos transitions’, while the RQA measures refer to chaotic- periodic state transitions. The laminarity measure, 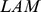, is defined as:



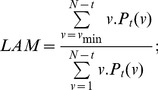
(8)


where 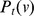
indicates the distribution of vertical line lengths.

Finally, the trapping time measure, 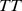 is given by:



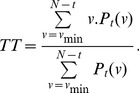
(9)


## Results and Discussion

We use one of the longest U.S. data series available for unemployment and output. The unemployment rates are taken from the U.S. Bureau of Labour while the output figures are taken from the U.S. Department of Commerce, Bureau of Economic Analysis. We emphasize we want to use U.S. data in this paper, as most of the studies written on this topic were performed with the help of U.S. data. The sample ranges from the first quarter of 1949 to the last quarter of 2010. We have opted for a quarterly frequency in order to ensure a high number of observations. The data were ensured to be stationary following the procedure in [33].

A first step in implementing quantitative recurrence analysis requires the determination of the key parameters, namely the embedding parameter 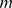, and the delay parameter 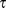. Recall that those two parameters were first encountered in equation (1) above. We must pay particular attention to the presence of noise which proves a distorting factor in computing the key parameters 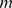
and 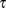. To aid in this, ‘mutual information’ is used in the case of the delay parameter and the ‘false nearest neighbours technique’ is employed when determining the embedding dimension. See also our brief discussion above equation (2).

It has been observed by [24] that a sufficiently large embedding would be sufficient to contain all relevant dynamics. In his application, he chooses an embedding of order 10 in order to take into account the high complexity of human dynamics. However, for the case of the lag he suggested the use of a lag 1 in the context of discrete economic data (financial data in his case).

We use the false nearest neighbours approach to determine the embedding dimension for both unemployment and output. We found an embedding dimension of 4 for each of the series. For the delay parameter, given the discussion in the literature, we simply choose a delay parameter 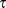 equal to 1.

### Recurrence plots

**Figure 1:**
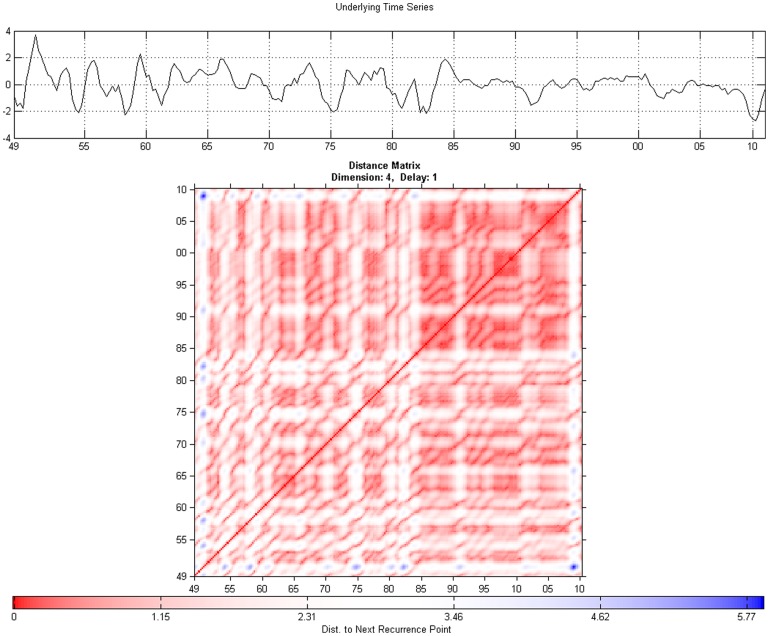
Unthresholded Recurrence Plot for output with Euclidean Distance.

**Figure 2:**
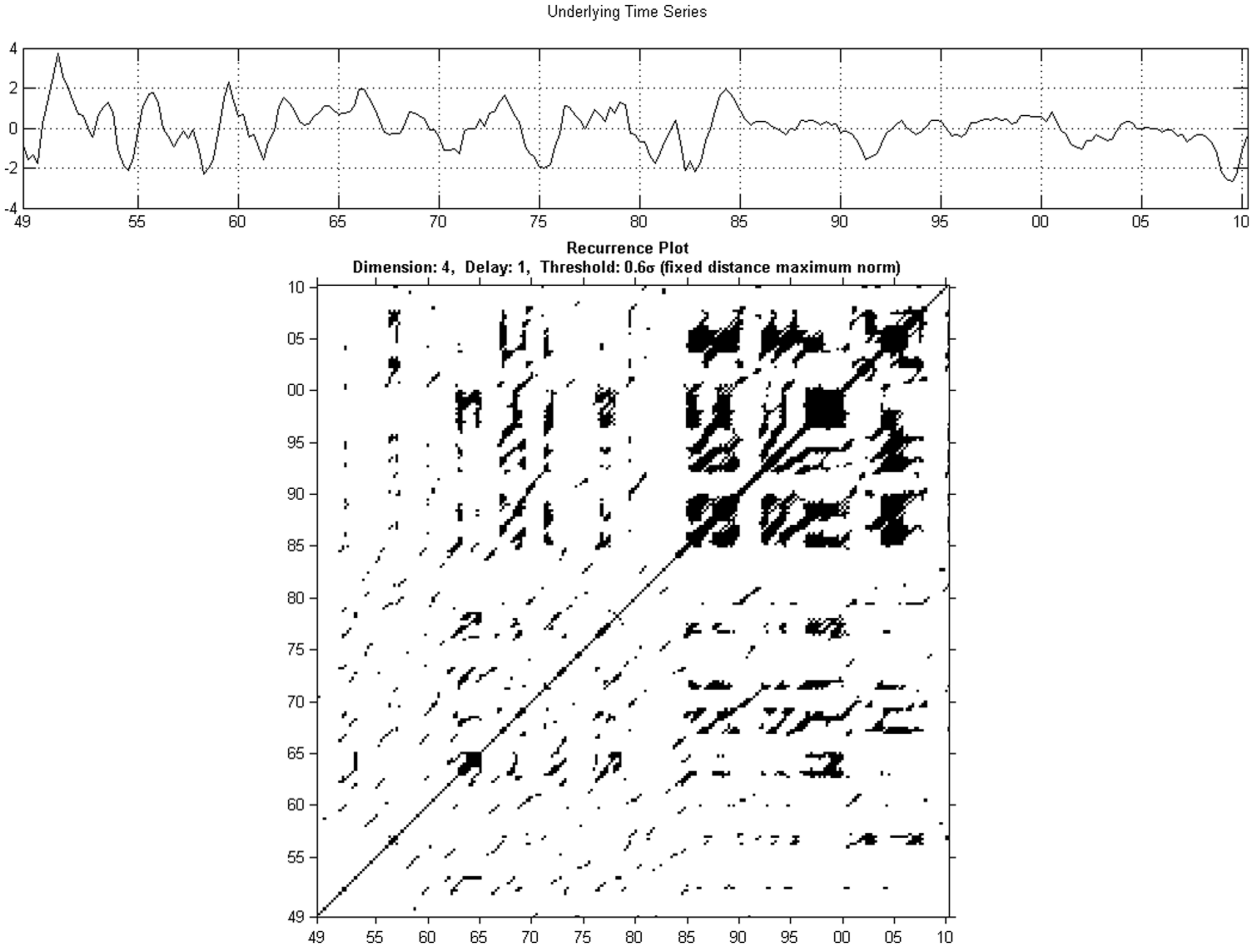
Thresholded recurrence plot for output with Euclidean distance and 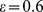
 **.**

**Figure 3:**
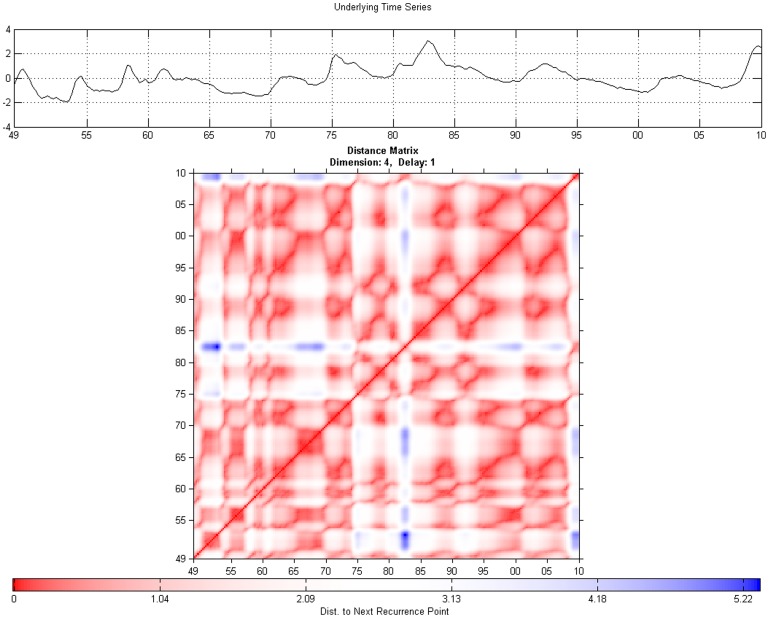
Unthresholded Recurrence Plot for unemployment rate with Euclidean Distance.

**Figure 4:**
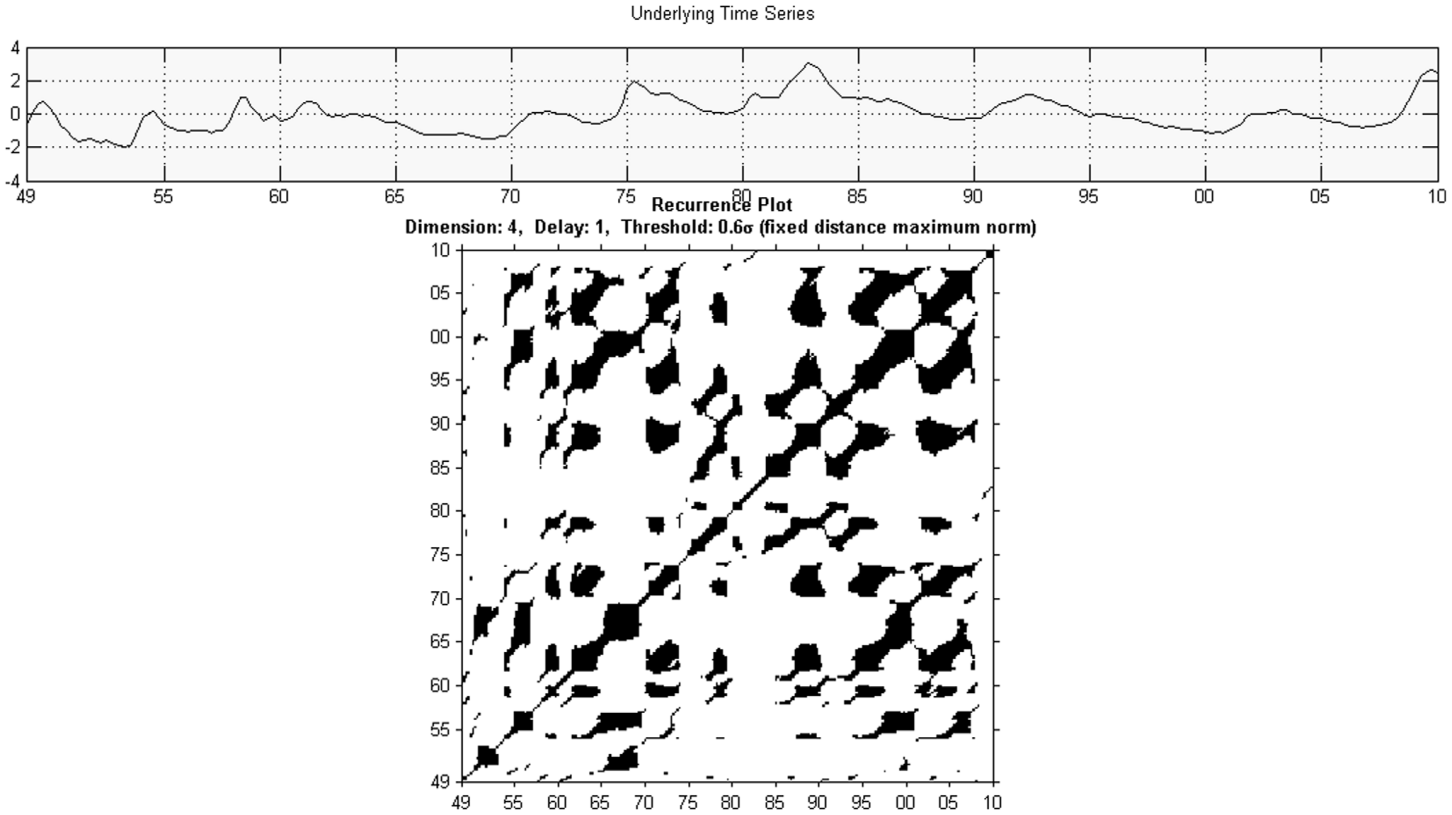
Thresholded recurrence plot for unemployment with Euclidean distance and 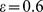
 **.**

**Figure 5:**
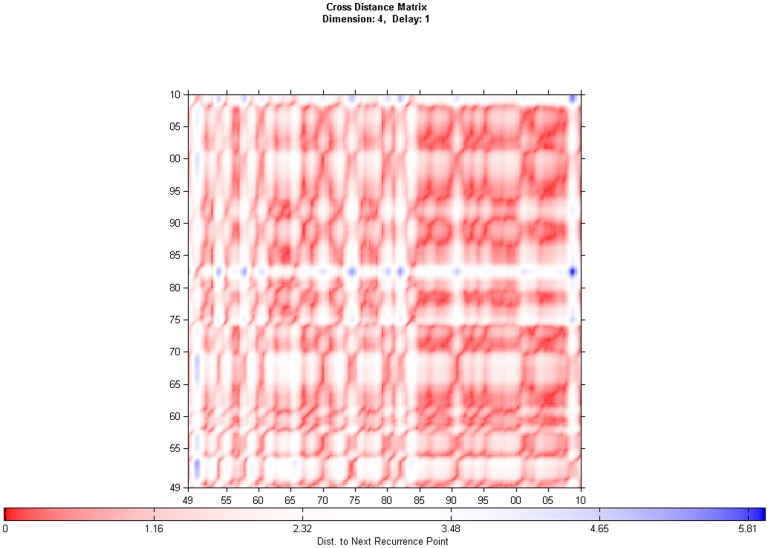
Untresholded cross recurrence plot for unemployment and output with Euclidean Distance 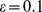
 **.**

**Figure 6:**
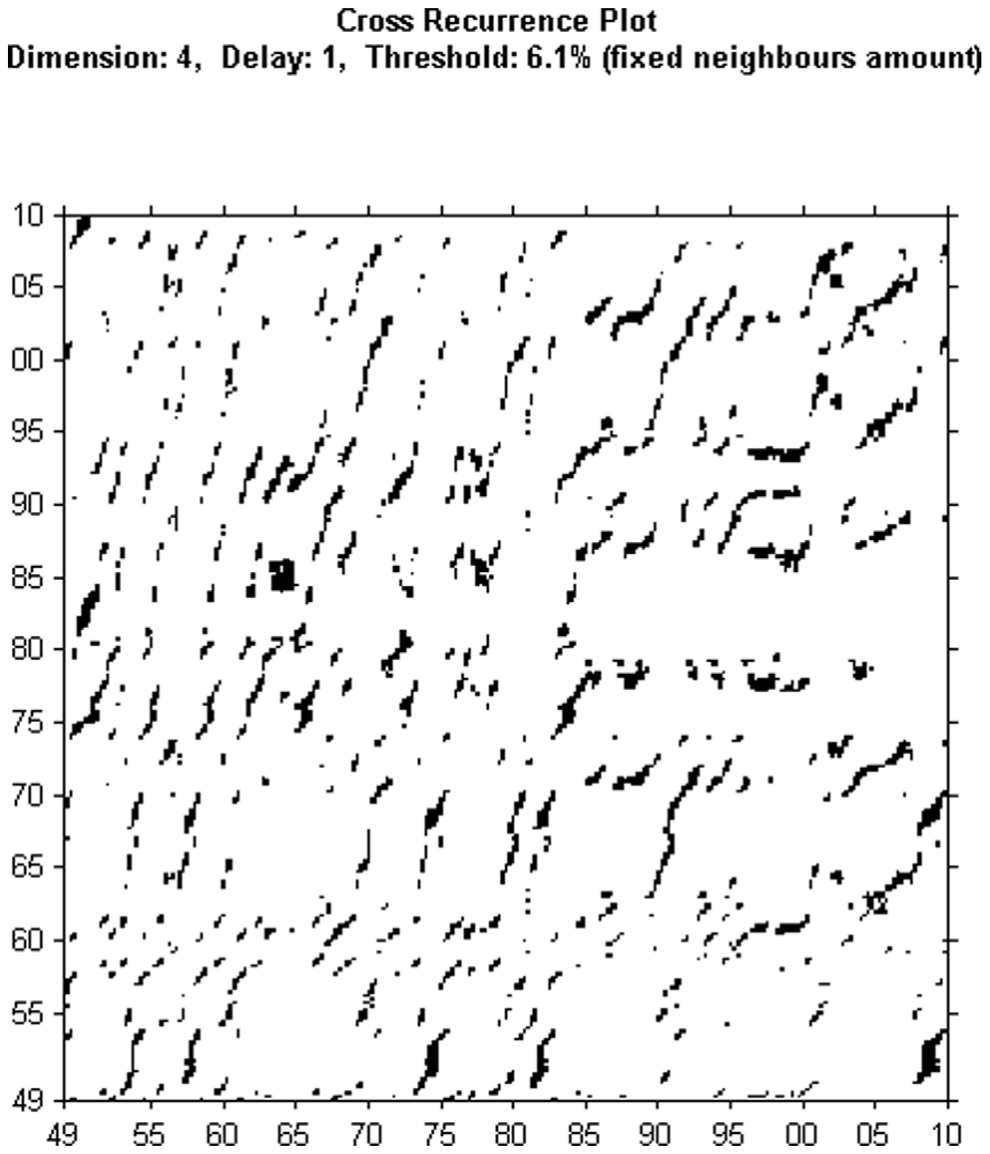
Thresholded cross recurrence plot for unemployment and output with Euclidean distance and 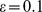
 **.**

Using the values determined above for the key parameters 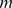
and 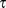, we perform a recurrence analysis of the two series (i.e. unemployment and output) we are interested in (see figures 1, 2, 3, 4, 5, and 6 below). We note that we are not using the log-change in output, but instead the annual growth of output as well as the unemployment rate. This is one of the main approaches in selecting the data, which is originally due to [24]. It is also used in [7] and [35]. Only recently in work starting with [36], do we see that the output gap can be used as a complementary alternative to the growth rate of output.

A third parameter must be determined, the radius parameter 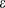, which also plays a crucial role in the results obtained through the recurrence plots. As [24] observes, choosing a too small value 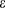 would lead into quantifying noise only, while a too high value would result in capturing values that are not really recurrent. One strategy [24] suggests is to compute the recurrence percentage for increasing values of the radius 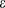
until a scaling region is reached. Other approaches in the literature are based on setting the radius based on the idea of obtaining reasonable recurrence rates (see for instance [28] and [33]). In a study on the Taiwanese unemployment rate, [29] used a value for 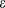 of 0.65. Since we will be studying unemployment rate series too, we will be using a value close to the one by [29], namely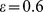.

Although the interpretation of the figures 1, 2, 3, and 4 (see below) is not straightforward, there are some indications in the literature on how one may read such figures. Eckmann et al. [22] provides for a very intuitive account on how to read some of the plots. Marwan [30] classifies patterns into large scale and small scale and according to his classification; he found four different types of large scale patterns or typologies.

- Homogeneous typologies, characterizing white noise;
- Periodic typologies, when recurrence plots present diagonal lines and checkerboard structures, for the case of oscillating systems;
- Drift typologies, when there is fading in the upper left and lower-right corners, for systems which are not stationary;
- Disrupted typologies, for extreme (and rare) events, when white bands are present, indicating transitions.

The second class, of small scale structures, can be characterized through the following characteristics:

- Single, isolated points, for rare states;
- Diagonal lines, when the system visits the same region of the phase space at different moments;
- Vertical horizontal lines, when the system either does not change or it changes slowly. This phenomenon is also an indicator of intermittency.

Looking at the recurrence plot figures for unemployment and output, figures 1, 2, 3, and 4, we can clearly reject the idea of random processes as the recurrence plots are not exhibiting a homogeneous typology.

We find several white vertical bands that make transitions between different block characterizing periods when the system behaved in a similar way. These bands correspond basically to the more severe recessions from 1973–1975 and 2009 and they can indicate also transitions in the dynamics. The patterns indicate a process which is to a degree predictable. A changing process occurs after 1975.

Figures 5 and 6 show the results from applying cross recurrence to both unemployment and output series. The dynamics of unemployment and output, as evidenced from the recurrence plots in figures 1, 2, 3, and 4, are again presented in figures 5 and 6. We note that the evidence suggested by figure 6 points to additional unusual patterns, as underlined by the ‘S’ shapes of segments. This might indicate some possible modifications in the relationship at lag levels. These features would be hard to uncover using standard econometric approaches (we thank two referees of this paper for this important insight). We observe again white bands corresponding to the recessions in 1973–1975 and 2009–2010. They mark again transition periods between two periods with similar dynamics. Within these periods, the dynamics are rather similar.

These results indicate that the recent literature on the output-unemployment relationship that stressed nonlinearities, time-dependency or state switching is indeed correct. However, our figures go even further, stressing the presence of a degree of predictability and the possibility of transition periods corresponding to the recessions in 1973–1975 and 2009–2010. Such results could be further tested using techniques from econometrics.

We note that the possibility of a changing nature of the business cycle is acknowledged in the economic literature. For example, starting with the early 1980s, the economic literature points to the emergence of the Great Moderation, a period of lower volatility that is characterized by specific dynamics, as recessions are smaller and lower in intensity. This period ended with the Great Recession, the last global economic and financial crisis. A key reference to the Great Moderation and how the business cycles changed after 1985 is the paper by [37]. A more general perspective on the historical changes in business cycles is provided for by [17]. There are other recessions, like the ones in the 1970s that are also known to have marked the transition between different types of business cycles. The literature acknowledges that, in general, the macroeconomic relationship between different variables (as is the case of output and unemployment) may change during severe downturns. Thus, we think we can link the evidence through recurrence plots with the idea of a changing nature of business cycles, since through recurrence plots we can identify the changing dynamics of a time series.

### Quantitative recurrence analysis

**Figure 7:**
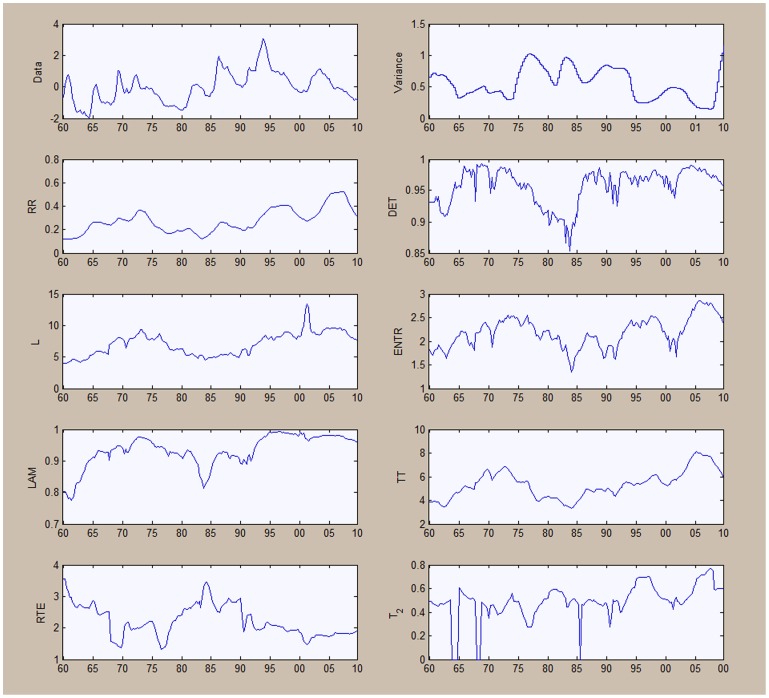
Quantitative recurrence analysis for unemployment Series.

**Figure 8:**
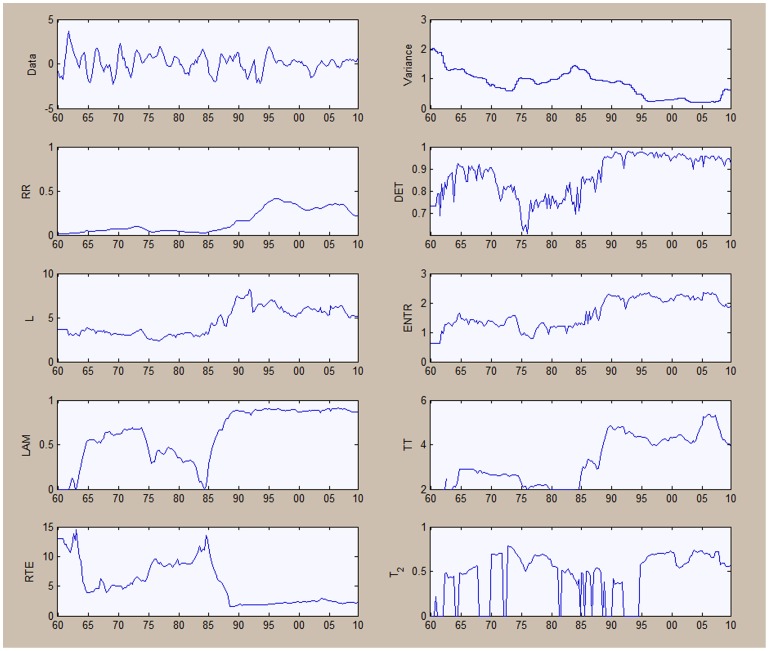
Quantitative recurrence analysis for output.

Before doing a cross analysis, we analyse each series using a windowed quantitative recurrence analysis. We use again an embedding dimension of 4 and a delay parameter of 1. The radius 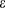
is chosen to be 0.6, while the minimum size for diagonals and vertical lines is chosen to be 2. The moving window is set at 48 periods which, given our quarterly frequency, is equivalent to 12 years. Figure 7 shows the results for the unemployment series, while the results for output are shown in figure 8.

The results can be read while keeping in mind the output obtained from the application of recurrence plots in figures 3 and 4. In Figure 7, when looking at the recurrence rate, we see a lowering of the recurrence rate corresponding to the two already evidenced recessions, from the mid-1970s and 2009–2010. The degree of predictability, as evidenced through the 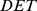
output, is also decreasing during this period. The results here are similar to those obtained from the recurrence plots: the unemployment series is characterized by a degree of predictability, with two transition periods.

The results in figure 8 from the recurrence plots are largely confirming established macroeconomic facts. The variance of output has largely fallen after the 1980's corresponding to the phenomenon called the ‘Great Moderation’. We notice an increase in the recurrence rate of production after period 120 (around 1975) implying an increased level of predictability, which can also be seen from the 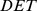
output. The recurrence rate was generally much lower than that for unemployment during the same period, suggesting a stochastic process for output between the 1950s and mid-1970s. The line length increases also within the second period, with line lengths around 6–7.

**Figure 9:**
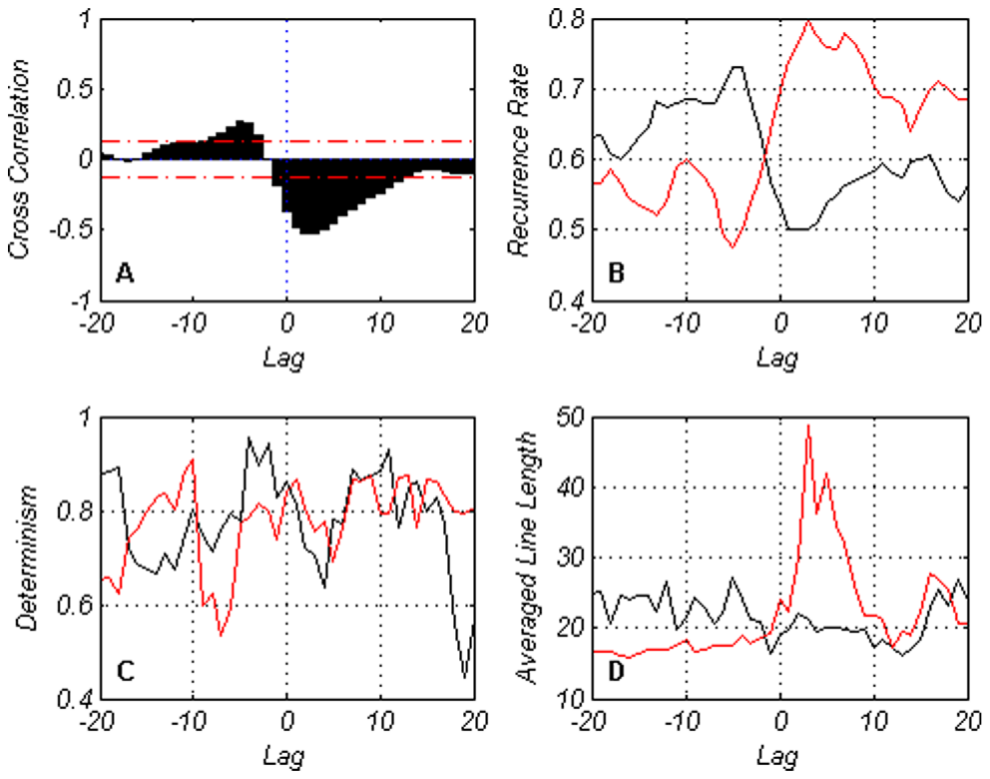
Quantitative recurrence diagonal analysis for output and unemployment. Red lines correspond to the negative relationship while black lines to the positive one.

**Figure 10:**
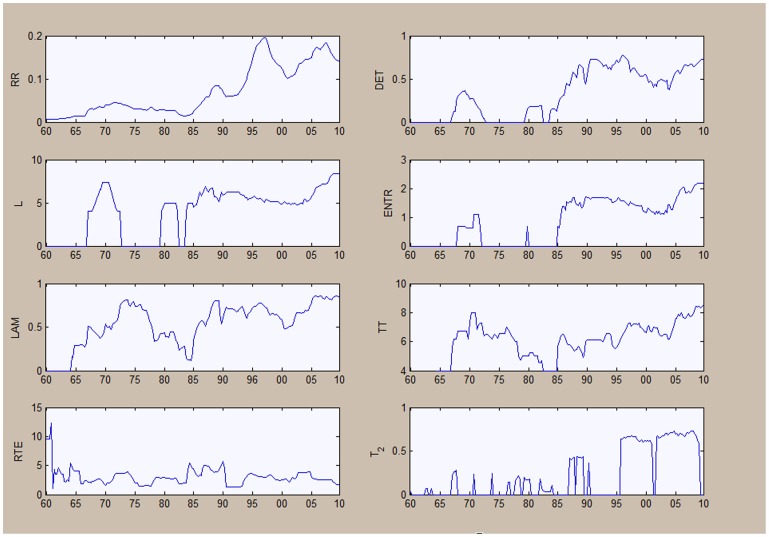
Cross quantitative recurrence analysis for output and unemployment.

We discuss in the next two figures (figures 9 and 10), two measures of cross analysis based on quantitative recurrence analysis: quantitative recurrence diagonal analysis in figure 9 and cross quantitative recurrence analysis in Figure 10.

The quantitative recurrence diagonal analysis is based on an embedding dimension of 4 with a delay parameter of 1. The window is 40, corresponding to a 10 year period, with 5 years for negative lags, and 5 years for positive lags. The negative relationship is the strongest one, as expected, and it is slightly biased toward positive lags, suggesting a delayed response by unemployment. The same delay can be found in the peaks reached by the recurrence plots and average line length. We recall the words of [3] (please see the introduction section of our paper) who claimed that the responsiveness of unemployment to output changes was very much a dynamic phenomenon.

The relationship, based on figure 9, can be considered as characterized by a degree of predictability, as evidenced from the recurrence rate and determinism rate. Moreover, it also shows there is a more complex picture emerging of the relationship between unemployment rate and output. It may be questioned whether such complex relationship could be easily uncovered with standard econometric techniques.

In figure 10 we further investigate the relationship between output and unemployment using quantitative recurrence analysis based on an embedding dimension of four; a delay parameter of one; minimum vertical and diagonal lines of two and a radius distance 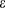
of 0.6. We use a sliding window of 48 periods (12 years).

The results are influenced by the individual dynamics of the series. The recurrence rate increases after the mid-1970s, with the 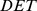
measure increasing also after this period. This signals an increased degree of predictability in the relationship of the series. The average line lengths increased after the ‘70s implying more pronounced common dynamics. We also noticed an increase in the overall complexity of the dynamics, evidence through the 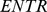
measure.

## Conclusions

In conclusion, we analysed the dynamics of output and unemployment in the United States. We considered as well the relations between output and unemployment using recurrence plots and quantitative recurrence analysis. We were able to isolate patterns in the dynamics of unemployment and output which, we believe could not be otherwise isolated using standard econometric tools. The series are found to be characterized by a degree of predictability, with periods of dynamic discontinuity corresponding to the large recessions, like the mid-1970s recession or the 2009–2010 recession. We thank one of the referees of this paper for emphasizing that recessions can be better termed as ‘dynamic discontinuities’.

These findings are in line with the main results found from research carried out during the last decades on understanding the relationship between unemployment and output. This research has pointed out nonlinearities, state switching and dynamic relations. In this paper, we go even further, by pointing to a degree of predictability and the possibility of a dynamic discontinuity in different states during the big recessions. Although we do not quantify the relationship, the findings here can be used to expand our understanding of the output -- unemployment relationship. However, it needs to be said that the potential evidence of degrees of predictability should be taken with caution. Marwan [34] makes the following comment on the 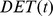
measure in equation (5): ‘High values of 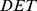
might be an indication of determinism in the studied system, but it is just a necessary condition, not a sufficient one.’ He indicates that even for non-deterministic processes it is possible to find longer diagonal lines with as consequence higher than warranted 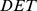
values.
